# Electroacupuncture Promotes the Survival of the Grafted Human MGE Neural Progenitors in Rats with Cerebral Ischemia by Promoting Angiogenesis and Inhibiting Inflammation

**DOI:** 10.1155/2021/4894881

**Published:** 2021-10-07

**Authors:** Juan Li, Luting Chen, Danping Li, Min Lu, Xiaolin Huang, Xiaohua Han, Hong Chen

**Affiliations:** ^1^Department of Rehabilitation Medicine, Tongji Hospital, Tongji Medical College, Huazhong University of Science and Technology, Wuhan, Hubei, China; ^2^Department of Rehabilitation Medicine, General Hospital of the Yangtze River Shipping, Wuhan, Hubei, China

## Abstract

Stem cells have the potential as a regenerative therapy for cerebral ischemia by improving functional outcomes. However, cell transplantation has some limitations, including a low rate of the grafted cell survival. There is still a major challenge of promoting the harmonious symbiosis between grafted cells and the host. Acupuncture can effectively improve the functional outcome after cerebral ischemia. The present study evaluated the therapeutic effects and explored the mechanism of combined medial ganglionic eminence (MGE) neural progenitors differentiated from human embryonic stem cells (hESCs) with electroacupuncture (EA) in a bilateral common carotid artery occlusion (2VO) rat model. The results showed that EA could promote the survival of the grafted MGE neural progenitors differentiated from hESCs and alleviate learning and memory impairment in rats with cerebral ischemia. This may have partially resulted from inhibited expression of TNF-*α* and IL-1*β* and increased vascular endothelial growth factor (VEGF) expression and blood vessel density in the hippocampus. Our findings indicated that EA could promote the survival of the grafted MGE neural progenitors and enhance transplantation therapy's efficacy by promoting angiogenesis and inhibiting inflammation.

## 1. Introduction

Ischemic cerebrovascular disease is a leading cause of mortality and disability worldwide after heart disease and cancer, lacking effective therapeutic methods [1]. In addition to causing hemiplegia, aphasia, swallowing disorders, etc., cerebral ischemia can also lead to a selective and delayed pyramidal neuronal death in the hippocampus and impair cognition function [2, 3]. Rapid vascular recanalization is a relatively effective therapy. However, some patients cannot get timely access to effective treatment because the window for vascular recanalization is very short [4] and often results in intracranial hemorrhage [5]. Some drugs proven effective in animals could not reach desired therapeutic benefit in the clinic [6, 7]. Therefore, further investigation on effective treatments and interventions against cerebral ischemia is urgently needed.

Recently, different kinds of stem cells have been transplanted to treat central nervous system diseases, such as stroke [8], spinal cord injury [9, 10], and Alzheimer's disease (AD) [11]. MGE (medial ganglionic eminence) is a structure of the ventral forebrain during embryonic development. A study found that in the absence of NKX2.1 (the main marker of MGE), cholinergic septohippocampal projection neurons and large subsets of basal forebrain cholinergic neurons fail to develop, causing severe deficiencies in learning and memory [12]. MGE neural progenitors can be used as a cell source to treat learning and memory impairment. Cell therapy can supply a sufficient number of cells for transplantation, but there is a major challenge in promoting the harmonious symbiosis between grafted cells and the host. After a cerebral ischemic injury, most grafted cells die as a result of malignant changes in the ischemic focus and the surrounding microenvironment; for example, inflammation or immune response, trophic factor withdrawal, oxidative stress, excitotoxicity, hypoxia, and apoptosis [13–16]. Hence, to a certain extent, the unfriendly microenvironment restricts the effect of cell transplantation therapy. Therefore, improving microenvironment and promoting the survival of MGE neural progenitors may be one of the strategies to enhance the transplantation effect and improve the cognitive impairment after cerebral ischemia.

Electroacupuncture (EA) delivers electrical stimulation to acupuncture points through acupuncture needles. Several studies have shown that EA can effectively improve neural function recovery after cerebral ischemia. The potential mechanisms include prevention of inflammatory and oxidant stress [17], suppression of apoptosis [18], and promotion of angiogenesis [19]. Additionally, a systematic review and meta-analysis indicated that its mechanism positively correlates with endogenous neurogenesis, in which EA therapy can promote the migration and differentiation of neural stem cells (NSCs) [20]. Studies have confirmed the vital role of electroacupuncture in treating central nervous system diseases combined with stem cell transplantation [21, 22]. Therefore, EA could improve the host's brain's microenvironment with cerebral ischemia and promote the survival of grafted MGE neural progenitors to improve the cognitive impairment. This article will focus on promoting angiogenesis and inhibiting inflammation after cerebral ischemia to verify the above hypothesis.

## 2. Materials and Methods

### 2.1. Human Embryonic Stem Cell (hESC) Culture and Neuronal Differentiation

The hESCs (hM3Dq-KORD, Passages 25-50; WiCell Research Institute) were cultured on a feeder layer of irradiated mouse embryonic fibroblasts (MEFs) using hESC medium, which included DMEM/F12 (Hyclone, C11330500BT), 20% knockout serum replacer (Gibco, 10828028), 1x GlutaMAX™ Supplement (Gibco, 35050061), 1x MEM Nonessential Amino Acid Solution Supplement (Gibco, 11140050), 0.1 mM *β*-mercaptoethanol (Sigma-Aldrich, M3148), and 8 ng/mL basic fibroblast growth factor (PeproTech, 10018B). The cells were expanded every six days.

**Figure 1:**
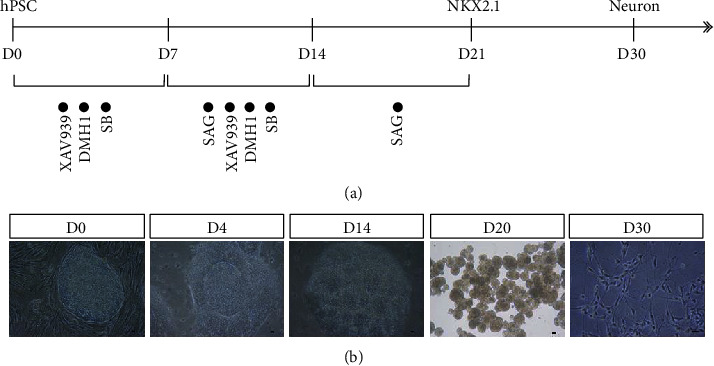
Differentiation of hESCs into MGE neural progenitors *in vitro*. (a) Flowchart of differentiation induced by the dual SMAD inhibition differentiation method. (b) Cell morphology at different stages of differentiation. Scale bars = 50 *μ*m.

MGE neural progenitors were differentiated by the dual SMAD inhibition differentiation protocol with MEFs as feeder layer, as described previously [23]. Briefly, the hESCs were cultured in neural differentiation medium, including 50% DMEM/F12 (Gibco, 11330032), 50% Neurobasal (Gibco, 21103049), 1x MEM Nonessential Amino Acid Solution Supplement, 1x N2 (Gibco, 17502048), 2 *μ*M SB431542 (Selleck, S1067), 2 *μ*M DMH1 (Selleck, S7146), and 2 *μ*M XAV939 (Stemgent, 040046) for seven days. The stem cells were differentiated into neuroepithelia in this process. Then, for ventralization, 0.5 *μ*M SAG (Sigma-Aldrich, 566660), a sonic hedgehog activator, was added at days 8-14 and the cells differentiated into NKX2.1 and Foxg1 coexpressing MGE neural progenitors. On day 14, the cells were digested and expanded as free-floating neural spheres for six days. On day 21, the neural spheres could be dissociated for transplantation or *in vitro* analysis. For *in vitro* analysis, the progenitor cells were spread onto polyornithine/laminin-coated coverslips with neurobasal medium with BDNF (10 ng/mL; PeproTech), GDNF (10 ng/mL; PeproTech), IGF1 (10 ng/mL; PeproTech), and cAMP (1 *μ*M, Sigma-Aldrich) (Figure 1).

### 2.2. Animals and Bilateral Common Carotid Artery Occlusion (2VO) Model

SPF-grade adult male Sprague-Dawley (SD) rats, weighing 250–280 g, were purchased from the Center of Experimental Animals, Tongji Medical College, Huazhong University of Science and Technology, Wuhan, China. All the procedures were approved by the Animal Care and Use Committee of the Tongji Medical College, Huazhong University of Science and Technology, Wuhan, China. SD rats were housed at 25 ± 2°C under a 12 h light/dark cycle with food and water ad libitum. The bilateral common carotid artery occlusion (2VO) model was generated as described previously [24]. Animals were divided randomly into five groups: (1) sham operation group (sham), (2) 2VO group (2VO), (3) 2VO combined with culture medium transplantation group (2VO+Vehicle), (4) 2VO combined with MGE neural progenitor transplantation group (2VO+Cell), and (5) 2VO combined with MGE neural progenitor transplantation and EA group (2VO+Cell+EA).

### 2.3. Electroacupuncture Therapy

**Figure 2:**
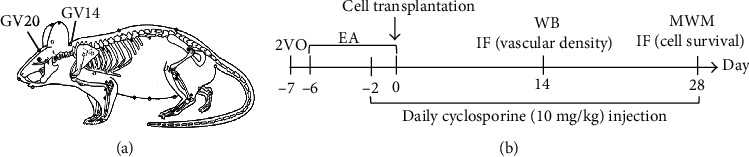
(a) Schematic diagram of GV20 and GV14 acupoints in the rat. (b) Flowchart of the experimental design.

The rats in the 2VO+Cell+EA group received EA treatment. The location of the “Baihui” (GV20) and “Dazhui” (GV14) acupoints was described previously [24] (Figure 2(a)). Acupuncture needles (0.3 mm diameter) were horizontally inserted at a depth of 10 mm into the GV20 and perpendicularly at 5 mm into GV14. Then, the electrical stimulation was delivered using a G6805-II electroacupuncture therapeutic apparatus (Shanghai Medical Electronic Apparatus Co., China), with a continuous current at 2 Hz for 20 min daily. The stimulation intensity was set according to the visible light facial muscle twitching. The entire treatment period was seven days starting from the first day after 2VO to the cell transplantation day (Figure 2(b)).

### 2.4. Cell Transplantation

Cell transplantation was performed seven days after 2VO as described previously [25]. In brief, MGE neural progenitors were dissociated with Accutase and prepared at approximately 1 × 10^5^ cells/*μ*L in the medium. The rats were anesthetized and fixed on the stereotaxic device. Next, the skull was exposed, and a small hole was drilled on per hemisphere. A total of 2.5 *μ*L cell suspension was transplanted into per side of hippocampus site (coordinates: anterior-posterior (AP) = −4.0 mm, medial-lateral (ML) = ±3.0 mm, and dorsal-ventral (DV) = −3 mm; 0 reference point for AP and ML at bregma, 0 reference point for DV at skull surface at the target site), according to the stereotaxic coordinates given in the rat atlas of Paxinos and Watson [26]. The injection time was continuous 5 min, and the needle was left for 5 min before removal to prevent cells from overflowing the needle track. All rats were injected subcutaneously with 10 mg/kg cyclosporine A, two days before transplantation and subsequently daily.

### 2.5. Laser Doppler Flowmetry

The cortical cerebral blood flow (CBF) was assessed using a laser Doppler flowmetry (MoorVMS, England). Rats were anesthetized and placed in a stereotactic apparatus. A burr hole (3 mm in diameter) was made through a midline scalp incision, with an electric drill on the right frontoparietal region (anterior-posterior (AP) = −1.0 mm, medial-lateral (ML) = −5.0 mm) to set a placement device for a contact probe. The CBF was quantified before and after the ligation of the common carotid arteries. Calculated perfusion was expressed as a percentage ratio of postischemic to the preischemic brain to account for variables.

### 2.6. Tissue Processing

Rats were deeply anesthetized with 10% chloral hydrate (3 mL/kg) and perfused with 4% paraformaldehyde (PFA). The brains were quickly dissected out and soaked in 4% PFA for at least 12 h. The rat brain tissues were subjected to gradient dehydration in 20% and 30% sucrose sequentially. The brain specimens containing transplantation sites (from 3.0 to 5.0 mm behind the bregma) were embedded in optimal cutting temperature compound and cut into 30 *μ*m coronal tissue sections with a frozen tissue slicer. The tissue sections were collected further in tissue cryopreservation fluid for immunohistochemical analysis.

### 2.7. Immunofluorescence Staining

Immunofluorescence staining was performed *in vitro* on coverslip cultures or frozen brain tissue sections as previously described [27]. Briefly, cells were fixed in 4% PFA for 20 min (frozen tissue sections did not require this step), washed with phosphate-buffered saline (PBS), and then blocked by QuickBlock™ Blocking Buffer for Immunol Staining (Beyotime, P0260) for 30 min at room temperature. The primary antibodies were added and incubated overnight at 4°C. Primary antibodies included mouse anti-Nestin (Millipore, MAB5326, 1 : 1000), rabbit anti-PAX6 (BioLegend, 901301, 1 : 1000), goat anti-SOX1 (R&D Systems, AF3369, 1 : 1000), rat anti-HOXB4 (DSHB, RRID: AB-2119288, 1 : 100), rabbit anti-Foxg1 (Abcam, Ab18259, 1 : 500), mouse anti-NKX2.1 (Millipore, MAB5460, 1 : 500), rabbit anti-GABA (Sigma-Aldrich, A2052, 1 : 1000), goat anti-CHAT (Millipore, AB144P, 1 : 300), mouse anti-neuronal class III *β*-tubulin (Tuj1; BioLegend, 801201, 1 : 2000), mouse anti-microtubule-associated protein-2 (Map2; Sigma-Aldrich, M1406, 1 : 1000), mouse anti-human nuclei (HuNu; Millipore, MAB1281, 1 : 500), goat anti-mCherry (Biorbyt, RRID: AB-2687829), and rabbit anti-NeuN (Millipore, ABN78, 1 : 1000). The corresponding fluorescent-conjugated secondary antibodies were applied for 1 h, and the nuclei were stained with DAPI (Boster, AR1177).

### 2.8. Vascular Density Analysis

Vascular density in the hippocampus was evaluated with immunofluorescence staining on the 14^th^ day after grafting. Coronal brain sections (30 *μ*m) on every six sections were used for vascular density analysis. The stained vasculature was observed within the hippocampus using an Olympus FV1000 confocal microscope (Olympus, Japan, ×10 objective). A lectin-positive vessel separated from adjacent vessels was counted as one vessel. The number of these vessels was added to the number of vascular branch points (number of vessel bifurcations) to obtain the total number of vessels, as described previously [28]. Five fields for each section, randomly distributed, were imaged for statistical analysis, with at least three independent replication tests. An investigator blinded to the group manually quantified vascular density, and the data were expressed as the number of stained vessels per 0.36 mm^2^ under ×10 objective.

### 2.9. Microscopical Analysis and Cellular Quantification

As described previously [25], all nuclei of grafted cells were identified in the presence of the human-specific nuclear marker (HuNu) immunostaining. HuNu^+^/DAPI^+^ double-labeled cell counting was performed on every six sections on the 28^th^ day after grafting [29], and images were captured using an Olympus FV1000 confocal microscope (Olympus, Japan, ×40 objective). The numbers of HuNu^+^/DAPI^+^ double-labeled cells were counted on the images by an investigator blinded to the group using ImageJ software (NIH, Bethesda, Maryland, USA), and the data were expressed as the number of positive cells per area under ×40 objective. Five areas were randomly selected for each section, with at least three independent replication tests.

### 2.10. Western Blot Analysis

Under deep anesthesia, hippocampus brain tissue, including the grafting area, was immediately dissected on ice. After centrifugation at 12,000 rpm for 15 min, total protein was isolated by homogenization, and the supernatant was collected. The protein concentrations were estimated by BCA assay. The total protein was separated using sodium dodecyl sulfate-polyacrylamide gel electrophoresis (SDS-PAGE). After electrophoresis, it was transferred onto polyvinylidene difluoride (PVDF) membranes, blocked in milk, and incubated at 4°C overnight with primary antibodies: rabbit anti-VEGF (Affinity, AF5131, 1 : 1000), rabbit anti-TNF-*α* (CST, 8184S, 1 : 1000), and rabbit anti-IL-1*β* (CST, 12703S, 1 : 1000), followed by incubation with appropriate horseradish peroxidase-conjugated secondary antibodies. Blots were detected using the ECL Western Blotting Substrate, and the intensity of the protein band was quantified using Gel-Pro Analyzer 4.0 software (Media Cybernetics, USA).

### 2.11. Morris Water Maze Task

Morris water maze (MWM) task was used to evaluate hippocampus-dependent spatial learning and memory. Briefly, a circular water tank (150 cm in diameter and 50 cm in deep) was filled with water (23 ± 2°C) to a depth of 21 cm. A circular submerged platform (15 cm in diameter and 20 cm in height) was placed underwater and in the target quadrant center. Several visual cues were displayed on the wall of the test room. In the place navigation phase, rats were subjected to four trials per day for four consecutive days. Each trial lasted either until the rat found the platform or for 60 s. The starting point was changed for every trial. The rats were allowed to rest on the platform for 20 s after each trial. On the 5^th^ day, the platform was removed for a 60 s spatial probe trial to test rats' spatial memory. The latency to find the submerged platform, the dwell time in the target quadrant, and the swimming paths were recorded automatically using a computer-based image analyzer MWM tracking system MT-200 (Chengdu Technology & Market Co., Ltd., Chengdu, Sichuan Province, China).

### 2.12. Statistical Analysis

SPSS 22.0 software (IBM Corporation, Somers, New York, USA) was used for statistical analysis. To assess the normal distribution of data, the Shapiro-Wilk test was employed (*P* > 0.05). Data were analyzed for multiple comparisons by one-way analysis of variance (ANOVA) followed by Tukey's multiple comparison tests among different groups. When comparing two groups at two time points (CBF values), two-way analysis of variance (ANOVA) was implemented. When comparing two groups at the same time point, Student's *t*-test was implemented. All data were expressed as mean ± standard error of the mean (SEM). *P* value < 0.05 was considered statistically significant.

## 3. Results

### 3.1. Human MGE Neural Progenitors Were Efficiently Generated from hESCs *In Vitro*

During the culture process, small molecules SB431542, DMH1, XAV939, and SAG were applied to promote hESC differentiation into neural progenitor cells. Morphologically, neural tube structures appeared on the 4^th^ day of differentiation, and several rosettes, the typical structure of the neuroepithelium, appeared on the 14^th^ day. After being expanded as free-floating neural spheres, MGE neural progenitors were obtained on the 21^st^ day. The cells were then adherently cultured at this stage, and neurons were obtained after ten days (Figure 1(b)).

**Figure 3:**
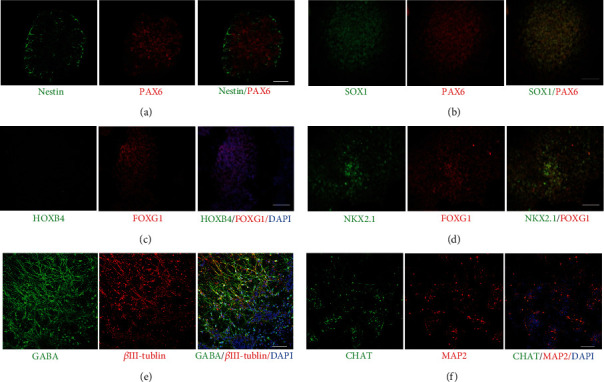
Generation of neural progenitors for transplantation and the neural markers of MGE neural progenitors during the process of differentiation. (a) Representative images were immunoreactive for neuroepithelium marker PAX6 and NSC marker Nestin on day 4. (b) Coimmunostaining SOX1 and PAX6 at day 10. (c) Differentiated hESCs expressed forebrain marker, FOXG1, but no spinal cord marker, HOXB4. (d) Coexpressing MGE neural progenitor marker NKX2.1 and FOXG1 on day 14. By 49 days in culture, neurons were positive for (e) GABA and (f) CHAT. Scale bars = 50 *μ*m.

In the presence of several small-molecule inhibitors, neuroepithelium marker PAX6 and NSC marker Nestin began to appear on the 4^th^ day (Figure 3(a)). On the 10^th^ day, another marker of the neuroepithelium, SOX1, was observed (Figure 3(b)). Additionally, the forebrain marker, FOXG1, but no spinal cord marker, HOXB4, was observed (Figure 3(c)). Meanwhile, the cells colabeling MGE marker NKX2.1 and FOXG1 appeared on the 14^th^ day (Figure 3(d)) and reached a peak on the 21^st^ day. MGE neural progenitors eventually differentiated into GABAergic and cholinergic neurons on the 49^th^ day *in vitro* (Figures 3(e) and 3(f)).

### 3.2. The 2VO Model Showed a Decrease in Cerebral Blood Flow (CBF)

**Figure 4:**
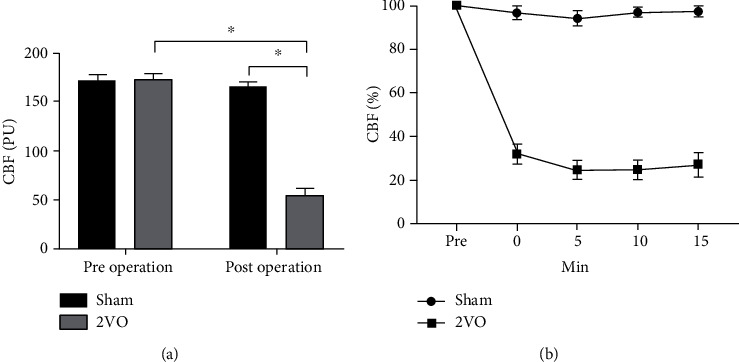
Laser Doppler monitoring of CBF in rats of the sham and 2VO groups. (a) Preoperative and postoperative blood flow values of the sham and 2VO group analysis of variance (two-way ANOVA, *F* = 275.036, *P* < 0.001). (b) The ratio of postischemic to preischemic CBF changes within 0–15 min before and after surgery. Values are mean ± SEM (*N* = 5 rats/per group). ^∗^*P* < 0.05.

The pre- and postoperative blood flow was monitored in rats of the sham and 2VO groups with laser Doppler. Before the operation, the mean baseline CBF values in the sham and 2VO groups were 171.9 ± 6.07 (PU) and 173.4 ± 5.26 (PU), respectively, with no significant difference. The CBF value in the 2VO group decreased to 54.9 ± 6.45 (PU), reaching the lowest within 5 min after the operation, and then maintained a low blood flow state, which indicated that 2VO rats suffered from persistent cerebral hypoperfusion (Figure 4).

### 3.3. EA Inhibited Inflammation of the Hippocampus in Rats of the 2VO+Cell Group

**Figure 5:**
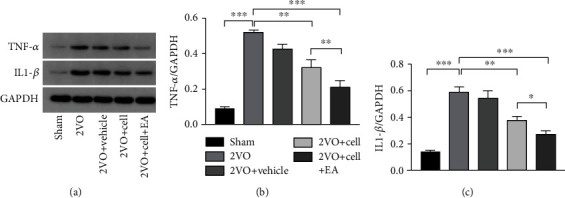
Effects of MGE neural progenitors and EA treatment on the expression of TNF-*α* and IL-1*β* in the hippocampus were evaluated by Western blot 14 days after grafting. (a) Gel electrophoresis of TNF-*α* and IL-1*β*. (b, c) Densitometric analysis of TNF-*α* (one-way ANOVA, *F* = 91.711, *P* < 0.001) and IL-1*β* (one-way ANOVA, *F* = 57.059, *P* < 0.001). Values are mean ± SEM (*N* = 5 rats/per group). ^∗^*P* < 0.05, ^∗∗^*P* < 0.01, and ^∗∗∗^*P* < 0.001.

Earlier studies suggested that postischemic inflammation plays a vital role in various stages of cerebral ischemic injury. Activated microglia leads to inflammasome-mediated interleukin- (IL-) 1*β* release, as well as tumor necrosis factor (TNF) production, which feedbacks into the inflammatory cascade by inducing cytokine and chemokine production in endothelial cells and astrocytes [30]. The inflammation leads to the deterioration of the local microenvironment, which is toxic to the host neural cells and is not conducive to the survival of exogenous grafted cells. It was reported that chronic cerebral hypoperfusion-induced cognitive impairment is associated highly with inflammation [31]. Therefore, proinflammatory cytokines were detected by Western blot analysis in this study to evaluate whether EA can inhibit inflammation, promote grafted cell survival, and alleviate cognition impairment. 2VO-induced inflammatory response in rats' hippocampus was demonstrated by the increased immunoreactivity of TNF-*α* and IL-1*β* compared to the sham group (*P* < 0.001). In the 2VO+Cell+EA group, immunoreactivity of TNF-*α* and IL-1*β* in the hippocampus decreased significantly compared with that of the 2VO+Cell group (*P* = 0.008 and *P* = 0.029), indicating that EA inhibited inflammation response of the hippocampus in rats of the 2VO+Cell group (Figure 5).

### 3.4. EA Promoted Angiogenesis of the Hippocampus in Rats of the 2VO+Cell Group

**Figure 6:**
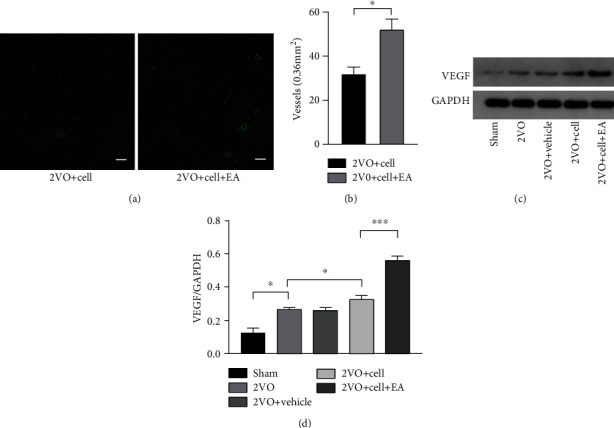
EA facilitates hippocampal angiogenesis in 2VO+Cell rats. (a) Immunofluorescence staining of blood vessels (green) in the hippocampus on the 14^th^ day after grafting. Scale bar = 100 *μ*m. (b) Quantification of vascular density in the 2VO+Cell and 2VO+Cell+EA groups. (c) Representative Western blots of VEGF on the 14^th^ day after grafting. (d) The densitometric analysis of VEGF level detected from the hippocampus in each group (one-way ANOVA, *F* = 13.574, *P* < 0.001). Values are mean ± SEM (*N* = 5 rats/per group). ^∗^*P* < 0.05 and ^∗∗∗^*P* < 0.001.

The key regulator of vascular development and homeostasis is the vascular endothelial growth factor (VEGF) [32]. In our study, Western blot analysis revealed a significant difference in VEGF protein expression in the hippocampus among the five groups. Two-vessel occlusion induced elevated VEGF protein expression in the hippocampus. Rats in the 2VO+Cell and 2VO+Cell+EA groups showed increased VEGF expression compared with the 2VO group at day 14 after transplantation. Compared with the 2VO+Cell group, VEGF protein expression was significantly higher in the 2VO+Cell+EA group rats (*P* < 0.001). Additionally, the vascular density of the cell grafting area in the hippocampus was detected by immunofluorescence staining. Vascular density in the 2VO+Cell +EA group rats (52.25 ± 6.50) was significantly higher than that in the 2VO+Cell group (32.75 ± 3.25) on day 14 after cell grafting (*P* = 0.017) (Figure 6). The above results indicated that EA promoted angiogenesis of the hippocampus in 2VO+Cell rats.

### 3.5. EA Promoted the Survival of the Grafted MGE Neural Progenitors in the Hippocampus

**Figure 7:**
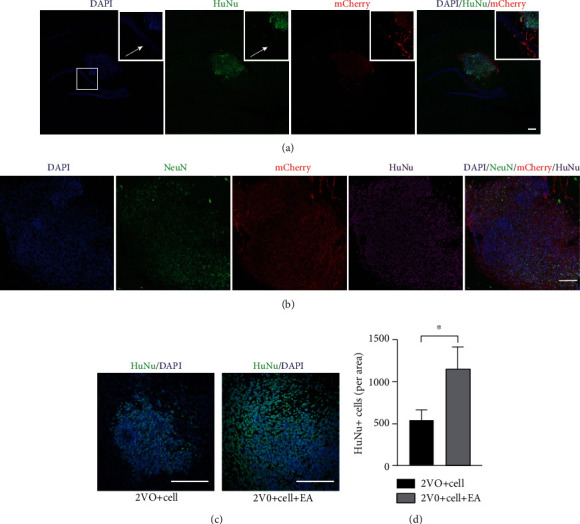
Effect of EA treatment on the survival of the grafted MGE neural progenitors in the hippocampus. (a) Fluorescence photomicrographs of HuNu^+^/mCherry^+^-positive cells from the 2VO+Cell+EA group. Scale bar = 200 *μ*m. (b) Some HuNu^+^/mCherry^+^-positive cells from the 2VO+Cell+EA group weakly expressed NeuN. Scale bar = 100 *μ*m. (c) Fluorescence photomicrographs of HuNu^+^-positive cells from the 2VO+Cell and 2VO+Cell+EA groups. Scale bar = 100 *μ*m. (d) Quantification of HuNu^+^-positive cells in the 2VO+Cell and 2VO+Cell+EA groups. Values are mean ± SEM (*N* = 5 rats/per group). ^∗^*P* < 0.05.

To better trace the grafted cells, hESCs modified by mCherry at AAVS1 site were used to differentiate into MGE neural progenitors for transplantation. Four weeks after transplantation, a large number of cells comarked with HuNu and mCherry were observed at the grafting area, indicating that grafted MGE neural progenitors differentiated from hESCs still survived in rats (Figure 7(a)). Some grafted MGE neural progenitors differentiated into neurons, weakly expressing NeuN (Figure 7(b)). Further analysis revealed that much more HuNu^+^ cells survived in the 2VO+Cell+EA group (1151 ± 260) than in the 2VO+Cell group (540 ± 123) in the grafting area (*P* = 0.021), indicating that EA promoted the survival of the grafted MGE neural progenitors in the hippocampus (Figures 7(c) and 7(d)).

### 3.6. MGE Neural Progenitor Transplantation Alleviated Learning and Memory Impairment in 2VO Rats and EA Enhanced Transplantation Therapy's Efficacy

**Figure 8:**
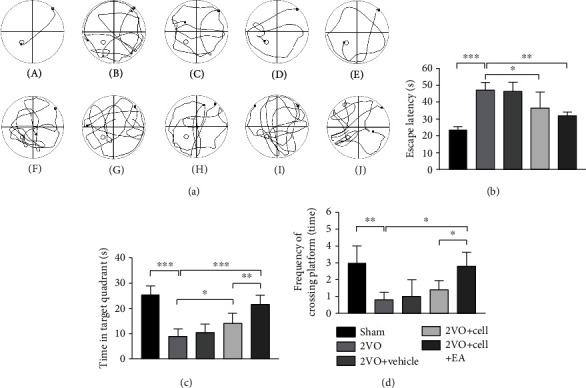
Comparison of the effects on spatial learning and memory test in rats from different groups. (a) (A–E) The swimming track of different groups of rats in spatial learning training; (F–J) the swimming track of different groups of rats in the spatial memory test. (A) and (F) from the sham group; (B) and (G) from the 2VO group; (C) and (H) from the 2VO+Vehicle group; (D) and (I) from the 2VO+Cell group; (E) and (J) from the 2VO+Cell+EA group. (b) Comparison of the average escape latencies of five groups in four days of spatial learning training (one-way ANOVA, *F* = 12.354, *P* < 0.001). (c) Comparison of the swimming time in target quadrant of five groups in the spatial memory test (one-way ANOVA, *F* = 22.159, *P* < 0.001). (d) Comparison of the frequency of crossing in the platform of five groups in the spatial memory test (one-way ANOVA, *F* = 8.281, *P* < 0.01). Values are mean ± SEM (*N* = 5 rats/per group). ^∗^*P* < 0.05, ^∗∗^*P* < 0.01, and ^∗∗∗^*P* < 0.001.

Four weeks after transplantation, the MWM task was used to assess the learning and memory ability of rats in each group, including spatial learning (Figure 8(a), A–E) and spatial memory test (Figure 8(a), F–J). After four days of spatial learning training, the escape latencies of rats in the 2VO group (47.2 ± 4.5 s) and the 2VO+Vehicle group (46.4 ± 5.4 s) were significantly longer than those in the sham group (23.7 ± 1.9 s) (*P* < 0.001). Compared with rats in the 2VO group, rats in the 2VO+Cell group showed a reduction in the escape latency (36.5 ± 9.5 s, *P* < 0.05) (Figure 8(b)) and showed increased time spent in the target quadrant (14.2 ± 3.9 s, *P* = 0.036) (Figure 8(c)), while the frequency of crossing in the platform was not different (1.4 ± 0.55, *P* = 0.759) (Figure 8(d)). Compared with the 2VO+Cell group, the escape latency of rats in the 2VO+Cell+EA group was shortened without significant difference (32.0 ± 2.2 s, *P* = 0.786) (Figure 8(b)). However, rats in the 2VO+Cell+EA group showed increased time spent in the target quadrant (21.6 ± 3.5 s, *P* = 0.019) and increased frequency of crossing the platform (2.8 ± 0.83, *P* = 0.047) compared with the 2VO+Cell group (Figures 8(c) and 8(d)). There were no significant differences between rats in the 2VO and 2VO+Vehicle groups in escape latency, time spent in the target quadrant, and the frequency of crossing the platform. The above results of the MWM task indicated that MGE neural progenitor transplantation alleviated learning and memory impairment in 2VO rats, and EA enhanced transplantation therapy's efficacy.

## 4. Discussion

Some neurological diseases are closely related to different types of neuronal damage, reduction, and death. For example, cognitive dysfunction in cerebral ischemia is related to cholinergic neuron loss in the hippocampus [33, 34]. Recently, with the development of differentiation technology, human pluripotent stem cells, including human embryonic stem cells (hESC) and human-induced pluripotent stem cells (hiPSC), can be induced to differentiate into specific neurons and glial cells *in vitro*, which provide a good source for cell transplantation therapy for these neurological diseases [35].

MGE (medial ganglionic eminence) is a structure of the ventral forebrain during embryonic development, having the main marker, NKX2.1. NKX2.1 is very important for the development of embryonic septal neuroepithelium and cholinergic neurons in the basal forebrain and the hippocampal cholinergic projection system. A study found that in the absence of NKX2.1, cholinergic septohippocampal projection neurons and large subsets of basal forebrain cholinergic neurons fail to develop, causing alterations in the hippocampal theta rhythms and severe deficiencies in learning and memory [12]. MGE neural progenitors are mainly differentiated into GABAergic neurons and cholinergic neurons *in vitro*, used as a cell source to treat learning and memory impairment. In this study, human embryonic stem cells were used to differentiate into MGE neural progenitors by combining induction using different small molecules for subsequent cell transplantation treatment. In the in vitro cell differentiation stage, a large number of NKX2.1-positive cells were observed on the 21st day. After another 28 days, the differentiated cells expressed GABA and CHAT, suggesting that the MGE neural progenitors obtained according to our differentiation protocol can further differentiate into GABAergic and cholinergic neurons *in vitro*. It thus lays the foundation for MGE neural progenitors grafting to improve the cognitive impairment caused by cerebral hypoperfusion in rats.

Neural stem cell transplantation can replace lost neurons and improve functional deficits because it has the inherent ability to differentiate into various cell phenotypes. Therefore, currently, cell transplantation has growing potential. However, there is still a long way to go, and some obstacles must be overcome before cell transplantation therapy can be actively used in the clinic. One of the major problems with cell transplantation is the low survival rate of grafted cells (5–20%). Most cell death occurs during the first few days after transplantation as a result of inflammation or immune response, trophic factor withdrawal, oxidative stress, excitotoxicity, hypoxia, and apoptosis [13–16]. Thus, for successful transplantation, improving the ischemic and hypoxic state of the hippocampus and inhibiting the inflammation in the grafted area, thereby improving the survival environment of grafted cells, may be an important strategy to improve the survival rate of grafted cells.

As an important treatment of Traditional Chinese Medicine, EA delivers electrical stimulation to acupoints through acupuncture needles. It has been recommended as a complementary therapy in stroke rehabilitation in Asian and Western countries. An increasing number of studies have shown that acupuncture can effectively improve the functional recovery of neurons after cerebral ischemia [36, 37]. The potential mechanisms include preventing inflammatory and oxidant stress, suppressing apoptosis, promoting angiogenesis, and endogenous neural regeneration. Since EA can promote angiogenesis and anti-inflammatory, it was combined with cell transplantation to study the survival of grafted cells in this study. Our findings showed that the 2VO model increased the expression of inflammatory factors, TNF-*α* and IL-1*β*, in the hippocampus, and the hypoperfusion produced by 2VO induced a slight increase in VEGF protein, consistent with the results of other studies [17, 28]. Compared with the 2VO+Cell group, EA inhibited TNF-*α* and IL-1*β* expression and increased the expression of VEGF protein and blood vessel density in the hippocampus of the 2VO+Cell+EA group. In the grafting area, several surviving cells coexpressing HuNu (for analysis of the survival of grafted cells, all nuclei of grafted cells were identified based on HuNu immunostaining) and mCherry were observed. These cells also expressed NeuN (fluorescence intensity was not as strong as the host cells), suggesting that they were gradually differentiating toward mature neurons. More HuNu^+^ cells were detected in the 2VO+Cell+EA group compared with the 2VO+Cell group through cell count analysis. Considering the above research results, we speculated that the higher survival of grafted cells in the 2VO+Cell+EA group was related to the EA effect on suppressing inflammatory and promoting angiogenesis.

As already established, the hippocampus plays a critical role in cognitive function [38]. As the basic structure or constitution, neurons of the hippocampus serve its local circuitry and process information. Since the loss of hippocampal neurons would induce cognitive deficits [39], cerebral ischemia could lead to selective and delayed pyramidal neuronal death in the hippocampal CA 1 area [40]. Thus, replenishing lost hippocampal neurons after ischemia may become an important treatment strategy to reduce cognitive damage. In the MWM task, compared with the rats in the 2VO group, the rats in the 2VO+Cell group showed a reduction in the escape latency and increased time spent in the target quadrant, indicating that learning and memory impairment in 2VO rats was alleviated. We speculated that the improved cognitive function was related to the replacement of partial dead hippocampal neurons by MGE neural progenitor transplantation. Compared with the 2VO+Cell group, the rats in the 2VO+Cell+EA group showed increased time spent in the target quadrant, and the frequency of crossing the platform was increased. This indicated that the ability of rats to learn and memorize in the 2VO+Cell+EA group was improved more significantly. Considering that more HuNu^+^-positive cells were observed in the 2VO+Cell+EA group in cellular quantification analysis, we speculated that EA facilitated the cell grafting effect on learning and memory impairment by promoting the grafted cells' survival in the hippocampus.

In summary, our study demonstrated that EA could promote the survival of the grafted MGE neural progenitors differentiated from human embryonic pluripotent stem cells and alleviated learning and memory impairment in rats with cerebral ischemia, which partially resulted from inhibited inflammation response and increased angiogenesis. This observation indicates that the combination of MGE neural progenitors with EA might be a promising approach to treat cognitive deficits after cerebral infarct. However, further studies and long-term observations are required to explore whether the grafted cells can differentiate into cholinergic and GABAergic neurons after maturation, their interaction and integration with host cells, and the formation of synaptic circuits to alleviate neural function deficits.

## Data Availability

The data used to support the findings of this study are available from the corresponding author upon reasonable request.
